# Integrative analysis of DNA methylomes reveals novel cell-free biomarkers in lung adenocarcinoma

**DOI:** 10.3389/fgene.2023.1175784

**Published:** 2023-06-16

**Authors:** Yifan Chen, Shanwu Ma, Chutong Lin, Zhipeng Zhu, Jie Bai, Zhongnan Yin, Yan Sun, Fengbiao Mao, Lixiang Xue, Shaohua Ma

**Affiliations:** ^1^ Department of Thoracic Surgery, Peking University Third Hospital, Beijing, China; ^2^ Institute of Medical Innovation and Research, Peking University Third Hospital, Beijing, China; ^3^ Cancer Center of Peking University Third Hospital, Peking University Third Hospital, Beijing, China; ^4^ Biobank, Peking University Third Hospital, Beijing, China; ^5^ Beijing Cancer Hospital and Institute, Peking University School of Oncology, Beijing, China

**Keywords:** lung adenocarcinomas, DNA methylation, circulating cell-free DNA, biomarker, GNA11

## Abstract

Lung cancer is a leading cause of cancer-related deaths worldwide, with a low 5-year survival rate due in part to a lack of clinically useful biomarkers. Recent studies have identified DNA methylation changes as potential cancer biomarkers. The present study identified cancer-specific CpG methylation changes by comparing genome-wide methylation data of cfDNA from lung adenocarcinomas (LUAD) patients and healthy donors in the discovery cohort. A total of 725 cell-free CpGs associated with LUAD risk were identified. Then XGBoost algorithm was performed to identify seven CpGs associated with LUAD risk. In the training phase, the 7-CpGs methylation panel was established to classify two different prognostic subgroups and showed a significant association with overall survival (OS) in LUAD patients. We found that the methylation of cg02261780 was negatively correlated with the expression of its representing gene GNA11. The methylation and expression of GNA11 were significantly associated with LAUD prognosis. Based on bisulfite PCR, the methylation levels of five CpGs (cg02261780, cg09595050, cg20193802, cg15309457, and cg05726109) were further validated in tumor tissues and matched non-malignant tissues from 20 LUAD patients. Finally, validation of the seven CpGs with RRBS data of cfDNA methylation was conducted and further proved the reliability of the 7-CpGs methylation panel. In conclusion, our study identified seven novel methylation markers from cfDNA methylation data which may contribute to better prognosis for LUAD patients.

## Introduction

Lung cancer is a leading cause of cancer-related deaths worldwide, accounting for 18% of all cancer-related deaths (Sung et al., 2021). The common histological subtype of lung cancer is non-small-cell lung cancer (NSCLC, accounting for 80% of lung cancer cases), in which LUAD is the major subtype and accounts for approximately 60% of NSCLC (Ferlay et al., 2019). LUAD is usually diagnosed at an advanced stage due to the minimal and non-specific early symptoms, and the limits of traditional screening methods such as chest radiography, sputum cytology, and computed tomography (Seijo et al., 2019). The average 5-year survival rate of LUAD is less than 20% (Siegel et al., 2021). Although the newly developed low-dose chest computed tomography (LDCT) screening technology has been introduced for the early detection of lung cancer, the increase in the survival rate seems to be limited (National Lung Screening Trial Research Team et al., 2011; Oken et al., 2011; Mazzone et al., 2021). Therefore, improved diagnostic techniques are of great significance for better outcomes of LUAD.

Considering the high costs of diagnostic techniques like LDCT, cost-effective and more sensitive non-invasive liquid biopsy methods have been taken into consideration. Great efforts have been made to identify potential blood biomarkers for LUAD, including genetic and epigenetic biomarkers (Spira et al., 2007; Ding et al., 2008; Ni et al., 2013; Silvestri et al., 2015). However, none of them have been fully established for the early diagnosis or prognosis of LUAD, due in part to the inconsistency between the characteristics of tissue and blood samples. Many biomarkers were found from tissue samples in previous studies, and they might probably fail when applied to blood samples (Hong and Kim, 2021). Therefore, it is necessary to identify novel biomarkers which are effective in liquid biopsy as well as in tissue biopsy.

In recent years, high-throughput genome-wide DNA sequencing technologies have rapidly developed and applied largely to discover cancer biomarkers. Many of them can be applied to the detection of circulating cell-free DNA (cfDNA) released by cancer cells in the blood. Especially the detection of cfDNA methylation levels can be achieved by whole-genome bisulfite sequencing (WGBS), reduced representation bisulfite sequencing (RRBS), and TET-assisted pyridine borane sequencing (TAPS) (Feng et al., 2019; Liu et al., 2019). Hence, it is possible to discover consistent methylation biomarkers directly from cfDNA in blood compared with that of gDNA in tissue.

**FIGURE 1:**
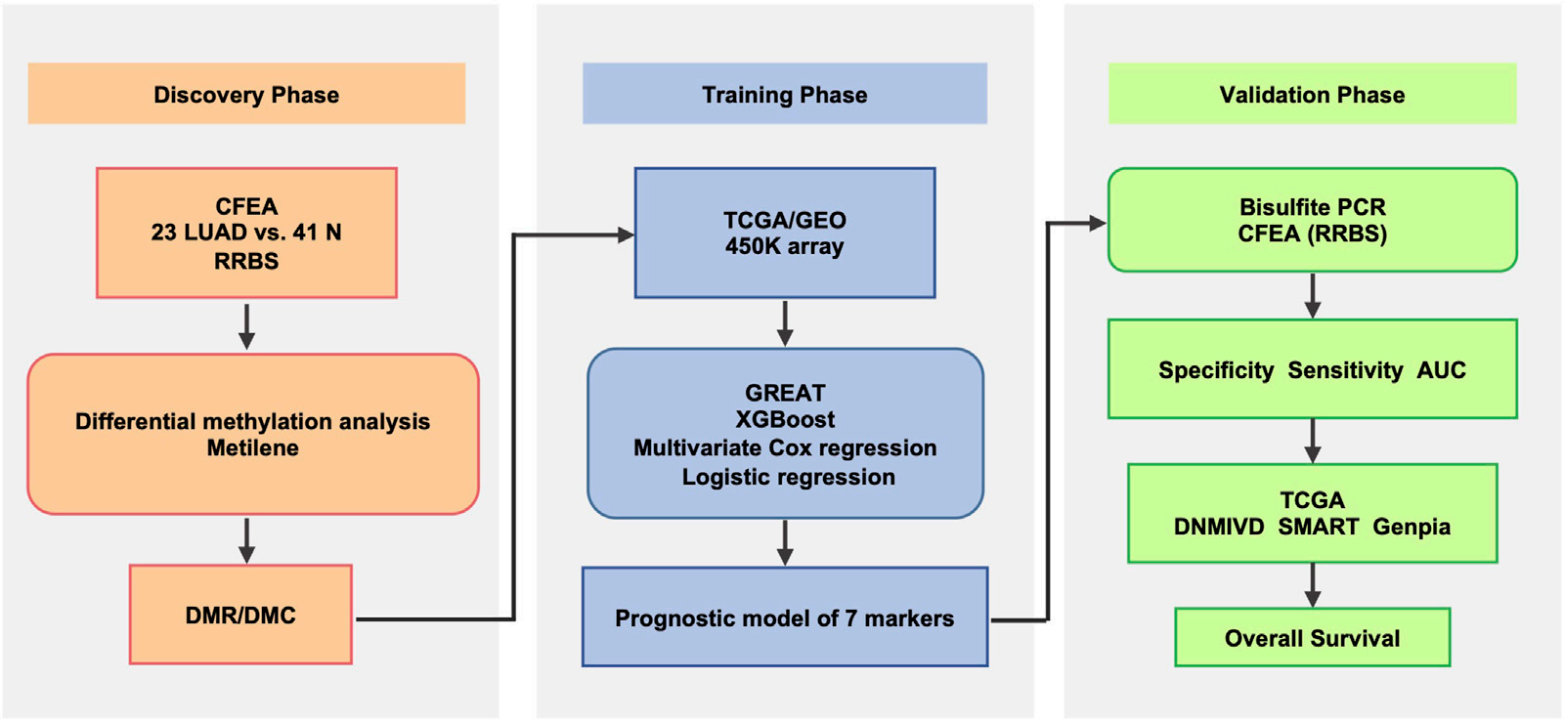
Workflow indicating study design. By comparing the genome-wide cfDNA methylation sequencing data between LUAD patients and healthy donors, 725 differentially methylated regions were found. Then XGBoost algorithm was performed to identify seven CpGs associated with LUAD risk. In the training phase, the 7-CpGs methylation prognostic model was established to classify different prognostic subgroups and showed a significant association with OS in LUAD patients. Based on bisulfite PCR, the methylation levels of 5 CpGs (cg02261780, cg09595050, cg20193802, cg15309457, and cg05726109) were further validated in tumor tissues and matched non-malignant tissues from 20 LUAD patients. Additionally, RRBS data of cfDNA samples from CFEA were analyzed to validate the methylation levels of the seven CpGs. CFEA, Cell-Free Epigenome Atlas; TCGA, the Cancer Genome Atlas; GEO, Gene Expression Omnibus; LUAD, lung adenocarcinoma.

In the present study, we aimed to identify clinical methylation biomarkers from cfDNA by using public RRBS data. By comparing the genome-wide cfDNA methylation sequencing data between LUAD patients and healthy donors, we found 725 differentially methylated CpGs. Then XGBoost algorithm was performed to identify seven CpGs associated with LUAD risk. In the training phase, the 7-CpGs methylation prognostic model was established to classify different prognostic subgroups and showed a significant association with OS in LUAD patients. Based on bisulfite PCR, the methylation levels of five CpGs (cg02261780, cg09595050, cg20193802, cg15309457, and cg05726109) were further validated in tumor tissues and matched non-malignant tissues from 20 LUAD patients. Validation of the seven CpGs with RRBS data of cfDNA methylation was further conducted and supported the reliability of the 7-CpGs methylation panel. Finally, a hypermethylation CpG locus (cg02261780) with its representing low-expression gene (GNA11) was also identified, which may contribute to the diagnosis and prognosis for LUAD patients (Figure 1).

## Materials and methods

### cfDNA methylomes

The cfDNA methylomes of lung cancer patients (*n* = 29) and healthy donors (*n* = 74) were downloaded from CFEA (Yu et al., 2020) (http://www.bio-data.cn/CFEA), which is a cell-free epigenome atlas for human diseases. These methylomes were detected by reduced representation bisulfite sequencing (RRBS). CfDNA Methylomes with covered CpGs < 5M and CpGs with sequencing depth <4 were removed. Finally, we collected 23 and 41 cfDNA methylomes from lung cancer samples and healthy donors. The basic information and statistics of RRBS data, including the mean value of mapped read depths and the percent of covered CpG, were included in Supplementary Table S1.

### DMC and DMR identification from cfDNA in CFEA

Metilene (v0.2-8) (Juhling et al., 2016) was used to call differentially methylated regions (DMRs) and sites (DMCs) from RRBS of cfDNA in CFEA. The command calling DMR was “metilene -M 300 -m 1 --minMethDiff 0.1 --threads 50 --mode 1 --mtc 2 --groupA normal --groupB cancer”, and the command calling DMC was “metilene -M 300 -m 1 --minMethDiff 0.1 --threads 50 --mode 3 --mtc 2 --groupA normal --groupB cancer”. DMCs fell into DMRs were used in the following analysis.

Trimmed RRBS reads were aligned to the reference genome using Bismark version 0.18.2 with default parameters. The Bismark_methylation_extractor program in the Bismark toolset was used to extract methylated CpG from aligned bam files. First, cfDNA methylomes with covered CpGs < 5M and CpGs with sequencing depth <4 were removed. Second, we employed metilene (v0.2-8) to identify DMR. Metilene is a segmentation algorithm to detect DMRs between single samples as well as in groups of samples (Juhling et al., 2016). As a distinguishing feature, it does not make assumptions on underlying distributions or background models and is applicable to WGBS as well as RRBS data without further parameter adjustments. In contrast to other approaches, metilene proposes a scoring model to find maximum intergroup methylation differences within a genomic interval of minimum length in combination with a nonparametric test. Based on a circular binary segmentation (CBS) (Siegmund., 1986; Olshen et al., 2004), metilene scans for pairs of change points within the mean difference signal (MDS), i.e., the difference of CpG-wise mean methylation level in the groups, delimiting a region with homogeneous methylation difference. Subsequently, intervals are tested for similarity using a two-dimensional Kolmogorov-Smirnov test (2D-KS test) (Fasano and Franceschini, 1987). Initially, the genome is presegmented to avoid calling DMRs containing long stretches without methylation information. These regions are recursively segmented until 1) a region contains less than a user-defined number of CpGs, or 2) no *p*-value improvement is achieved. Briefly, within a region [s, t], a window [a, b] is sought using the scoring function Zs, t (a, b), such that the MDS attains a maximal change. The algorithm checks for the existence of short methylation valleys embedded into longer differentially methylated regions and takes care of situations in which regions of differential up- and down-methylation are spatially adjacent. *p* values were adjusted by Benjamini–Hochberg (FDR). Finally, DMRs with FDR <0.05 and minimum mean methylation difference >= 0.1 were considered as significant DMRs.

### Identification of seven CpGs associated with LUAD risk and construction of the prognostic model

In the training phase, CpGs in the 725 LUAD DMRs and DMCs of the discovery cohort were trained with LUAD cohorts based on analyses with the Illumina Infinium HumanMethylation450 BeadChip collected from The Cancer Genome Atlas (TCGA-LUAD, https://cancergenome.nih.gov/) and the Gene Expression Omnibus (GEO) dataset. XGBoost algorithm (Wang et al., 2020; Wu et al., 2020) was performed subsequently to identify LUAD-specific CpGs with an important score >0.1. In our XGBoost model, we employed gradient tree boosting algorithm, a special form of gradient boosting machine and predicting, by combining the results of multiple weak learners. XGBoost classifier was introduced as the implementation of gradient tree boosting algorithm. XGBoost stands for eXtreme Gradient Boosting, which combines weak learners (decision trees (DTs)) to achieve stronger overall class discrimination. XGBoost learned a series of DTs to classify the labeled training data. Each DT comprises a series of rules that semi-optimally split the training data. Its sparsity-aware split search approach makes it suitable for our dataset where missing values commonly appear. Successive trees that ‘correct’ the errors in the initial tree were learned to improve the classification of positive and negative training examples. The overall survival analysis, Time-ROC curve, and multivariate regression analyses were performed to evaluate the prognostic value of the 7-CpGs methylation panel. The following stringent feature selection pipeline was used for constructing the prognostic model. 1) Standard deviation (SD) across all tumor samples should be >0.2. 2) FDR (Benjamini/Hochberg method) for every probe was calculated via univariate Cox regression in each cancer, and the probes with FDR <0.05 were remained for further filtration. 3) Log-rank test *p*-value of every probe for overall survival time among tumor samples should be <0.05. 4) Multivariate Cox regression was performed for the remained probes, and then stepwise regression was conducted, the probes of multivariate cox regression *p*-value >0.05 were removed from the feature set in each iteration. Finally, the remaining probes were used to fit the prognostic classifier. Python package lifelines (http://lifelines.readthedocs.io/en/latest/index.html) and Cox’s proportional hazard model were implemented in Cox regression analysis.

### Processing DNA methylation and gene expression data

For the correlation analysis of DNA methylation and gene expression, Entrez Gene ID was used to map sites assigned to a gene. Pearson’s correlation analysis was performed to obtain the correlation between DNA methylation and gene expression with SMART (http://www.bioinfo-zs.com/smartapp/) (Li et al., 2019). Those having an absolute Pearson correlation coefficient r) >= 0.3, 0.2–0.3, or 0.1–0.2 and *p* <= 0.05 were defined as strong, moderate, or weak correlations, respectively.

### RNA-seq data analysis

The R/Bioconductor package DESeq2 (Genome Biol. 2014; 15:550) was used to normalize raw RNA-seq read count from the LUAD cohort in TCGA. In total, 526 LUAD samples and 59 normal lung tissues were included in this analysis. Similar to our previous study (Li et al., 2021), the false discovery rate (FDR q-value) was calculated by adjusting *p*-values with the Benjamini–Hochberg method. Genes with FDR q-value <0.05 and |Log2 (Fold change) | > 1 were considered as DEGs.

### Clinical samples

Samples were collected from twenty patients pathologically diagnosed with LUAD. Matched adjacent normal tissues and primary tumors were obtained from all twenty patients. All patients were treatment-naive before tumor resection. The demographic and clinical characteristics of the patients were summarized in Table 1.

**TABLE 1:** The clinicopathologic features of validation cohorts.

Characteristics	Category
Gender	Male (n = 14), Female (n = 6)
Age	<60 (n = 4), ≥60 (n = 16)
Histological type	Adenocarcinoma (n = 20)
Tumor size	≤3 cm (n = 14), >3 cm (n = 6)
Stage	I/II (n = 10), III/IV (n = 10)
Lymph metastasis	N_0_(n = 7), N_1_/N_2_/N_3_ (n = 13)
Smoking habit	Never (n = 12), Smoker (n = 8)

### Bisulfite PCR

Genomic DNA of the tumor and adjacent normal tissues from LUAD patients were isolated using the QIAamp DNA Mini kit (Qiagen). One microgram of DNA was bisulfite-treated using the EZ DNA methylation Gold kit (Zymo Research). The bisulfate-treated and purified DNA was used for PCR or nested PCR. For bisulfite sequencing, the PCR products were gel extracted and ligated into a pCE2 TA/Blunt-Zero vector by using the TA cloning system (Vazyme). At least 10 separate clones were chosen for sequencing analysis. For bisulfite pyrosequencing, the PCR products were labeled with biotin and then conducted to pyrosequencing. PCR was conducted with EpiArt HS Taq Master Mix (Vazyme) following the manufacturer’s instructions. Bisulfite pyrosequencing was conducted with Pyromark Q48 Autoprep (Qiagen). Successful analysis showed that the percentage of samples with good quality was all 100%.

The primers for bisulfite PCR or nested PCR were designed by MethPrimer (Li and Dahiya, 2002) or PyroMark Assay Design 2.0, and were listed as follows:

cg02261780 (GNA11):

Outer Pair: OF: 5′TTA​GGT​TTT​GGG​GTA​GTA​GGG3’; OR: 5′AAA​CAA​TAA​ACA​CTA​TCT​AAA​AAC​ATC​TAT3′

Inner Pair: IF: 5′GGT​TTG​TAA​TTA​GGT​GGA​GTA3’; IR: 5′AAA​AAA​CTC​CTA​ACA​TAA​AAT​AAA​TAA​AAA3′ (374 bp; 58°C)

cg09595050 (PRDM8):

F:5′AAGTTTAGGGATTTGGGTGTGTAGG3′

R:5′CCTTCCCACTCCTATAAAAAACTATAAC3′ (88 bp; 55°C)

cg20193802 (GP1BB/SEPT5):

F:5′ATGAGGTTGTTTTGGGTTAGTTTGT3′

R:5′CCCCCAAAACCTAATCTCCCTA3’ (179 bp; 55°C)

cg15309457:

F:5′GGGGAGTGAAGAGGGTTTTTTATAGT3′

R:5′CAAAATATTTCCATCTAAAATCCTACAC3′ (253 bp; 55°C)

cg05726109 (GP1BB/SEPT5):

F:5′TAGTGGAGGGGATGGGTTAGGTA3′

R:5′CCCCACATACTTCCTCATCCTTC3′ (201 bp; 55°C)

The sequencing primers for bisulfite pyrosequencing were listed as follows:

cg09595050 (PRDM8): 5′GAG​GGT​TGG​AGT​TAT​T3’;

cg20193802 (GP1BB/SEPT5): 5′GGT​TTA​GAG​TGG​GTT​G3’;

cg15309457:5′GGG​TTT​TTG​ATA​GGT​GTT3’;

cg05726109 (GP1BB/SEPT5): 5′CCA​TAC​CAC​TAT​CCT​AAA​TCA​ATT3’.

### Statistical analysis

The statistical analysis was done by using Student’s t-test with Graphpad and *p* < 0.01 or 0.05 or adjusted *p* < 0.01 or 0.05 was considered significant. Outcome variables were tested for normality using the Kolmogorov-Smirnov test. Levene’s test was used to test variance equality. The ROC analysis was performed to determine the area under the curve (AUC) and was analyzed with Graphpad and SPSS v26. Survival analysis was performed using the log-rank test with DNMIVD (Ding et al., 2019) (http://119.3.41.228/dnmivd/index/) or Genpia (Tang et al., 2017) (http://gepia.cancer-pku.cn/detail.php).

## Results

### Identification of differentially methylated regions and sites in cfDNA in LUAD

**FIGURE 2:**
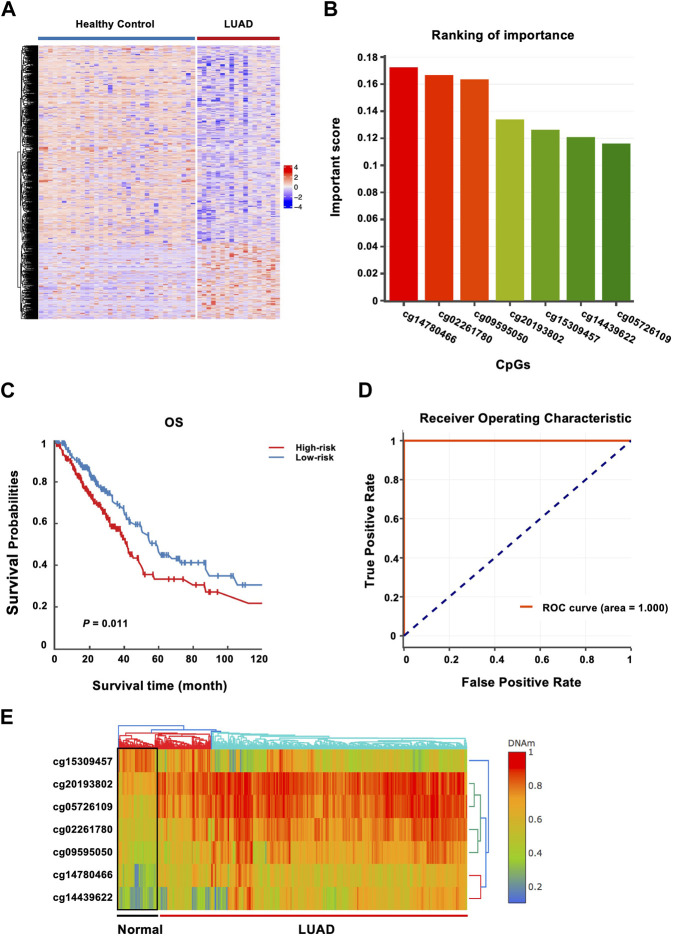
Identification of methylated genes associated with LUAD risk and construction of a prognostic 7-CpGs methylation panel. **(A)** Heatmap of the methylation levels of DMRs from CFEA datasets. The DMRs and DMCs between LUAD patients and healthy donors were analyzed with Metilene and data downloaded from CFEA, including cfDNA methylomes of LUAD patients (n = 29) and healthy donors (n = 74) by RRBS. CfDNA Methylomes with covered CpGs < 5M and CpGs with sequencing depth <4 were removed. 23 and 41 cfDNA methylomes from cancer samples and healthy donors were finally collected. **(B)** 7 CpG sites differentially methylated between 460 LUAD tissues and 32 adjacent normal tissues from TCGA and GEO cohorts were identified with the XGBoost algorithm in the training phase. DNMIVD was employed to build the diagnostic model based on the XGBoost algorithm, and whether the sample is normal, benign, or malignant was then predicted, and the basic information about the inputted CpGs was obtained, including methylation status and important scores. The important score for each CpG is calculated by XGBoost. The importance of the seven CpG sites was ranked by the important scores. **(C)** Survival analysis of the 7-CpGs methylation panel was conducted with a multivariate proportional hazards regression model with training cohort. **(D)** ROC curve analysis of the 7-CpGs methylation panel was conducted with Logistic regression with training cohort. **(E)** The interactive unsupervised hierarchical clustering and heatmap associated with the methylation profile of screened CpGs.

In the discovery phase, by comparing the genome-wide cfDNA methylation data of LUAD patients and healthy donors from the CFEA database, a total of 725 DMRs were identified. 154 of these CpG loci gained DNA methylation, whereas 571 loci were hypomethylated in LUAD (Figure 2A). GREAT (McLean et al., 2010) annotation analysis revealed that those LUAD-specific methylation changes often affected genes with roles in definitive hemopoiesis (adjusted *p =* 8.38E-8), negative regulation of Notch signaling pathway (adjusted *p =* 3.04E-6) and genetic imprinting (adjusted *P =* 1E-5).

### Building a prognostic 7-CpGs methylation panel

In the training phase, by combined analysis of DNA methylation profiles of LUAD tissues and adjacent normal tissues from the TCGA and GEO databases, the 725 DMRs identified from CFEA database were narrowed down to seven CpGs using the XGBoost algorithm (Figure 2B). The corresponding genes might serve as potential biomarker candidates with diagnostic and prognostic possibilities. The characteristics of the identified CpGs associated with LUAD risk were shown in Table 2.

**TABLE 2:** Characteristics of the 7-CpGs methylation panel.

CpG	Gene symbol	Group	Relation to island	Methylation status^a^	Score
cg14780466	GDF7	Body	Island	hyper	0.17,244,898
cg02261780	GNA11	Body	Island	hyper	0.16671449
cg09595050	PRDM8	5′UTR	Island	hyper	0.16359529
cg20193802	GP1BB/SEPT5	TSS1500/Body	Island	hyper	0.13393398
cg15309457	NA	NA	Island	hypo	0.12630065
cg14439622	GATA6	Body	Island	hyper	0.12089247
cg05726109	GP1BB/SEPT5	TSS1500/Body	Island	hyper	0.1161142

^a^ Methylation levels of the CpG locus in the datasets.

Then the seven CpGs were conducted to predict OS in the training cohort. We first performed survival analysis for each CpGs in TCGA, and we found that cg02261780 (adjusted *p =* 0.0306) and cg14439622 (adjusted *p =* 0.0131) are significant (Supplementary Table S2). Since this 7-CpG panel was identified by using a diagnostic model, the power of each CpG in prognosis may be different. So we next conducted multivariate proportional hazards regression model based on the combo of 7-CpGs, and the result showed that the 7-CpGs methylation panel was significantly associated with OS. The low-risk group was associated with increased OS times compared with the high-risk group (permutation *p =* 0.011) (Figure 2C), with DMC analysis in cell-free blood samples shown in Supplementary Table S3. Finally, the receiver operating characteristic (ROC) curve analysis showed that the methylation status of the 7-CpGs methylation panel had statistically significant power to distinguish people with low-risk from high-risk (Figure 2D). And the interactive unsupervised hierarchical clustering and heatmap associated with the methylation profile of screened CpGs were shown in Figure 2E. Finally, by further analyzing the prognostic ability of the 7-CpGs methylation panel in 10 cancer types with methylation data from tissue samples, we found that the panel also showed great prognostic roles in some cancer types, such as kidney renal clear cell carcinoma (KIRC), lung squamous cell carcinoma (LUSC), and thyroid carcinoma (THCA), in addition to LUAD. And when analyzing the diagnostic value of the 7-CpGs methylation panel, it exerted the highest diagnostic power in LUAD compared with other cancer types (Supplementary Table S6). Considering that these analyses were conducted with methylation data gained from tissue samples instead of blood samples, it could not be evaluated whether the 7-CpGs methylation panel could also be well applied to the blood samples of KIRC, LUSC, or THCA. Nevertheless, the diagnostic and prognostic power of the panel in LUAD was clear since the seven CpGs were discovered from the cfDNA methylation data of LUAD. Collectively, these results demonstrated the significant power of the 7-CpGs methylation panel for the diagnosis and prognosis of LUAD in liquid biopsy.

### Methylation of cg02261780 and the representing gene GNA11 in LUAD

**FIGURE 3:**
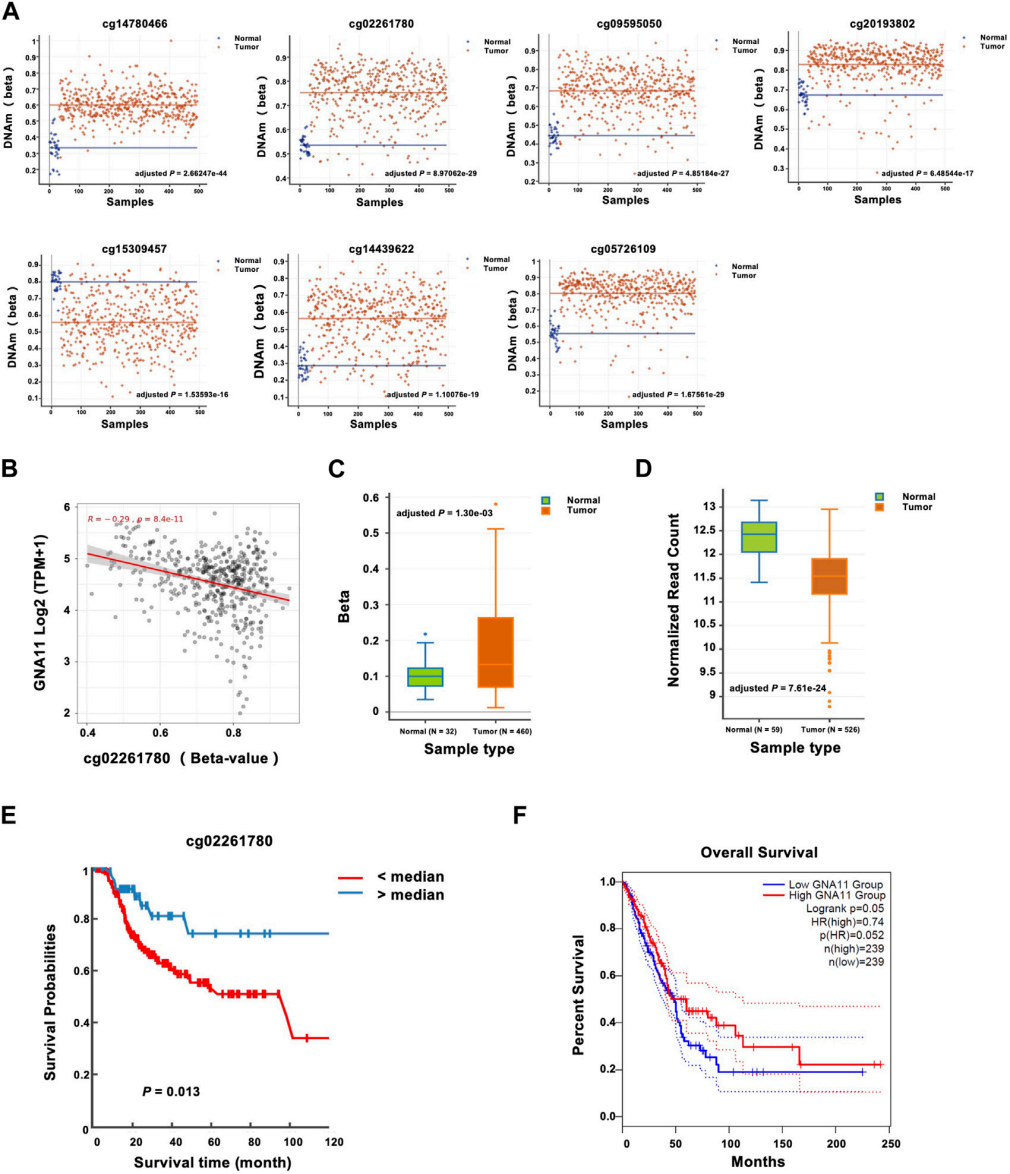
Prognostic significance of GNA11 expression and methylation in LUAD. **(A)** The methylation levels of cg14780466, cg02261780, cg09595050, cg20193802, cg15309457, cg14439622, and cg05726109 between normal and LUAD tissues were analyzed with DNMIVD, and the results were shown with the scatter plot. **(B)** The correlation between cg02261780 and GNA11 expression was analyzed with SMART across all transcriptome (Pearson coefficient r = −0.29; adjusted *p =* 8.4E-11). **(C)** The methylation levels of the GNA11 promoter between adjacent normal and LUAD tissues were analyzed with DNMIVD and were shown with the boxplot (N_Normal_ = 32, N_Tumor_ = 460, adjusted *p =* 1.30e-03). **(D)** The mRNA levels of GNA11 between adjacent normal and LUAD tissues were analyzed with DNMIVD, and the results were shown with the boxplot (N_Normal_ = 59, N_Tumor_ = 526, adjusted *p =* 7.61e-24). **(E)** Kaplan-Meier plots were produced by DNMIVD and showed the discriminatory power of cg02261780 methylation by the median of a *ß*-value (*p =* 0.013, log-rank test). **(F)** Survival analysis by Genpia showed the association of GNA11 expression levels with OS time (*p =* 0.05, log-rank test). The dashed lines indicated the 95% confidence interval information of the Cox proportional hazard ratio in the survival plot. The cohorts were analyzed from each website annotated above.

By characterizing the seven CpGs associated with LUAD risk, we found that the CpGs were consistently hypermethylated (cg14780466, cg02261780, cg09595050, cg20193802, cg14439622, and cg05726109) or hypomethylated (cg15309457) in LUAD tissues, which further confirmed the results of the bioinformatics analyses in plasmas (Figure 3A). Then we analyzed the correlation between the methylation of the CpGs and gene expression, and we found that among the seven CpGs, the methylation level of cg02261780 was negatively correlated with gene expression of GNA11 (Pearson coefficient r = −0.29; adjusted *p =* 8.4E-11) (Figure 3B, Supplementary Table S3), indicating a methylation-dependent transcriptional regulatory mechanism for GNA11. In addition, the methylation of the GNA11 promoter was also upregulated in the LUAD tissues than that in the normal tissues (Figure 3C). Opposite to the elevated methylation changes, GNA11 expression was downregulated in LUAD (Figure 3D).

Then the predictive role of cg02261780 methylation was further analyzed. The median of a *ß*-value of cg02261780 was used as the cut-off point to distinguish between the high-risk and middle-risk groups, and the KM curve could effectively distinguish the > median and < median groups (log-rank *p =* 0.013) (Figure 3E). On the other hand, based on GNA11 expression levels, patients could also be classified into two distinct prognostic subgroups, in which tumors exhibiting decreased GNA11 expression levels were associated with shorter OS time (log-rank *p =* 0.05) (Figure 3F).

Together these results indicated that GNA11 may serve tumor suppressive roles in the progression of LUADs, and may represent a novel biomarker for this disease. The integrated epigenetic and transcriptional assessment of GNA11 may be useful for optimizing the risk stratification of LUAD patients.

### Validation of seven CpGs by bisulfite PCR and RRBS

**FIGURE 4:**
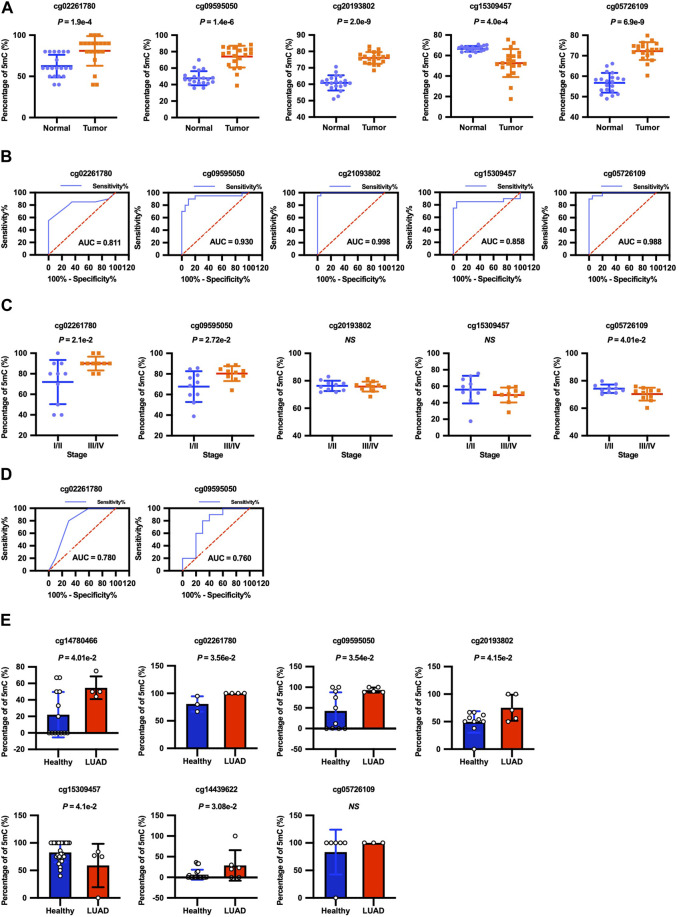
Diagnostic significance of cg02261780, cg09595050, cg20193802, cg15309457, and cg05726109 methylation levels. **(A)** The methylation levels of the cg02261780 (*p =*1.9E-4), cg09595050 (*p =* 1.4E-6), cg20193802 (*p =* 2.0E-9), cg15309457 (*p =*4.0E-4), and cg05726109 (*p =* 6.9E-9) loci in adjacent normal tissues and LUAD tumor tissues from 20 LUAD patients were detected by bisulfite PCR, and the results were shown with the scatter plot as mean ± SEM. **(B)** ROC curve analysis shows the sensitivity and specificity for discrimination between normal tissue with tumor tissue by the methylation levels of cg02261780, cg09595050, cg20193802, cg15309457, and cg05726109. **(C)** The methylation levels of the cg02261780, cg09595050, cg20193802, cg15309457, and cg05726109 loci in tumor tissues in 20 LUAD patients with different stages. **(D)** ROC curve analysis shows the sensitivity and specificity for discrimination between the early stage and advanced stage by the methylation levels of cg02261780 and cg09595050. **(E)** The cfDNA methylomes of lung cancers (n = 6) and healthy donors (n = 33), which were detected by RRBS, were downloaded from CFEA, and the methylation levels of these CpG sites were analyzed.

We next attempted bisulfite PCR on tumor tissues and matched non-malignant tissues from 20 LUAD patients to confirm the clinical roles of cg02261780, cg09595050, cg20193802, cg15309457, and cg05726109 methylation in LUAD. Consistent with the cfDNA data, the methylation levels of the cg02261780, cg09595050, cg20193802, and cg05726109 locus were indeed elevated between tumor tissues compared with normal tissues, while the methylation level of the cg15309457 locus was decreased in tumor tissues compared with normal tissues (Figure 4A). ROC curve analysis showed that the methylation levels of cg02261780, cg09595050, cg20193802, cg15309457, and cg05726109 all had statistically significant power to discriminate between normal tissue and tumor tissue (Figure 4B; Table 3). Therefore, the methylation levels of cg02261780, cg09595050, cg20193802, cg15309457, and cg05726109 could be potential diagnostic markers for LUAD.

**TABLE 3:** ROC curve analysis of the diagnostic efficiency of the five CpGs.

CpG	AUC	Sensitivity (%)	Specificity (%)	95% CI	*p*-Value E)
cg02261780	0.811	55	100	0.664–0.958	8.0–4
cg09595050	0.930	90	90	0.838–1.022	3.0–6
cg20193802	0.998	100	95	0.989–1.006	7.3–8
cg15309457	0.858	100	95	0.712–1.003	1.0–4
cg05726109	0.988	95	95	0.963–1.012	1.3–7

According to the progress of LUAD, we divided the patients into the early stage (stage I-II) and advanced stage (stage III-IV) groups. The methylation levels of cg02261780 and cg09595050 in patients with advanced stages were highly elevated compared with the patients in the early stages. And the methylation levels of cg05726109 in patients in advanced stages were decreased compared with the patients in early stages (Figure 4C). ROC curve analysis showed that the methylation levels of cg02261780 and cg09595050 had statistically significant power to discriminate between advanced stage and early stage. The area under the ROC curve of cg02261780 and cg09595050 was 0.780 and 0.760, with a sensitivity of 80% and 80%, specificity of 70% and 70%, and 95% CI of 0.566–0.994 (*p =* 0.0343) and 0.539–0.981(*p =* 0.0494), respectively (Figure 4D). These results suggested that the methylation levels of cg02261780 and cg09595050 might be used to discriminate between different stages of LUAD.

Additionally, we analyzed the methylation levels of the markers in the cfDNA with published data from CFEA. As indicated by Figure 4E, the methylation levels of cg14780466, cg02261780, cg09595050, cg20193802, cg14439622, and cg15309457 from cfDNA of LUAD patients and healthy donors were consistent with those in our discovery phase and training phase, which further supported the reliability of our 7-CpGs methylation panel.

## Discussion

In the present study, we used the whole genome methylation data in a discovery cohort and got 725 significant DMRs by comparing the methylation levels of cfDNA from LUAD patients and healthy individuals. Subsequently, we performed the XGBoost algorithm similar to our previous study (Wang et al., 2020) to identify seven CpGs associated with LUAD risk. We also reported a novel prognostic model for LUAD based on the DNA methylation levels of the seven CpGs by analyzing the association with OS. Among the seven CpG loci, we found that the methylation of cg02261780 and the expression of its representing gene GNA11 were negatively correlated and were associated with the OS of LUAD patients. The new seven CpGs methylation feature and hypermethylation of GNA11 exhibit particularly promising significance for LUADs. Finally, based on bisulfite PCR, the methylation levels of five CpG loci (cg02261780, cg09595050, cg20193802, cg15309457, and cg05726109) were further validated in tumor tissues and matched non-malignant tissues from 20 LUAD patients. DNA methylation levels of the five CpG loci have significant diagnostic value to discriminate normal tissue from tumor tissue. DNA methylation levels of the cg02261780 and cg09595050 loci could discriminate early stage from the advanced stage. The methylation levels of the markers in cfDNA samples with published data from CFEA further validated the reliability of our 7-CpGs methylation panel.

Of the seven methylation markers in the prognostic panel associated with LUAD risk, the methylation of cg02261780 and the expression of the related gene GNA11 demonstrated particularly promising significance for LUAD diagnosis and prognosis. GNA11, one of the Guanine nucleotide-binding protein subunit *a* (GNA) family members, can encode G-protein activating subunits binding to G protein-coupled receptors (GPCRs) and play central roles in cellular signaling transduction (Pierce, Premont, and Lefkowitz, 2002). G-proteins and GPCRs are ubiquitously expressed in various types of tissues and cells, and GNA11 plays an important role in multiple cell functions, including transcription, motility, and secretion (O'Hayre et al., 2013). In recent years, advances in whole genome sequencing technology have unearthed a previously unappreciated widespread role of GNA11 in melanomas (O'Hayre et al., 2013). GNA11 aberrations were found to exhibit the strongest association with ocular melanoma and appendiceal cancer across a range of malignancies (Van Raamsdonk et al., 2010; Danielli et al., 2011; Parish et al., 2018). Besides mutations, the GNA11 gene promoter was also found to be hypermethylated in hepatocellular carcinoma (HCC) and was proposed as a promising biomarker for diagnosis and targeted therapy (Livingstone et al., 2020). Together, these results indicated that epigenetic and transcriptional abnormalities in GNA11 were commonly implicated in the tumorigenesis of human cancer. However, the specific roles of GNA11 in regulating tumorigenesis and the relevant mechanisms still need further study, and whether GNA11 is regulated by the DNA methylation mechanism is still unknown (Larribère and Utikal, 2020). In the current study, we found that the methylation level of the cg02261780 locus was elevated in LUAD compared with normal tissue, and the methylation level was negatively correlated with GNA11 gene expression. Hypermethylation of the GNA11 probe cg02261780 was correlated with a poorer prognosis in LUAD patients.

Collectively, these results suggested that hypermethylation of the GNA11 probe cg02261780 downregulated GNA11 expression and was associated with poor survival outcomes in patients with LUAD, which offered new insights into the role of GNA11 in tumorigenesis. We also noticed that there was no GNA11 aberration observed in LUAD, suggesting that a methylation-dependent transcriptional regulatory mechanism for GNA11 plays an important role in the occurrence of LUAD, but not GNA11 aberration (Supplementary Figure S1). We further investigated the association between GNA11 and tumor stage, and found that the expression level of GNA11 in early and advanced stages was significantly lower than that in normal tissue (Supplementary Figure S2). However, no significant differences were observed at different tumor stage, indicating that dysregulation of GNA11 may mainly contribute to the tumorigenesis of LUAD, but not progression.

Evaluation of DNA methylation profiles is a promising strategy for the diagnosis and prognosis of not only lung cancer but also other cancer types, including breast cancer, colon cancer, liver cancer, and so on (Hao et al., 2017; Luo et al., 2021). And previous studies have revealed the advantages of cfDNA detection over traditional tumor biopsy (Wan et al., 2017). First, detection with cfDNA from blood sample is more convenient and non-invasive, which is more beneficial for clinical application. Second, we can find the most significant differential methylation sites in blood with cfDNA methylome. Third, plasma cfDNA can be used for dynamic monitoring of efficacy or prognosis. In addition, cfDNA might carry DNA methylation information from metastases, which is hard to acquire with tissue biopsy. Hence, it is possible to trace the metastatic state of LUAD patients with cfDNA methylation information. Finally, cfDNA can provide a full view of the molecular landscape of primary cancer, avoiding the bias with tissue biopsy caused by tumor heterogeneity. Therefore, great efforts have been made in discovering cfDNA biomarkers, especially cfDNA methylation markers. In most previous studies, DNA methylation markers were found by comparing the differential methylation regions between tumor tissue and normal adjacent tissue (Liggett et al., 2011; Langevin et al., 2012; Ooki et al., 2017; Xu et al., 2017; Vrba and Futscher, 2018). However, such a strategy might encounter a problem that tissue-originated DNA biomarkers might not be able to be detected in plasma since tumor-originated circulating tumor DNA (ctDNA) might only account for a minimal part of cfDNA (Pantel and Alix-Panabieres, 2019). Hence some high-risk methylation sites in tumor tissue might be missing in the ctDNA/cfDNA. In recent years, some studies have been trying to discover DNA methylation markers from cfDNA with high-throughput technologies to compensate for such inconsistency between cfDNA and gDNA of tumor tissue. For instance, novel cfDNA methylation markers for breast cancer were discovered with the joint analysis of methylation profiles from cfDNA and tumor tissues. And it was found that combining the DNA methylation characteristics with traditional diagnostic imaging can improve the current clinical practice for breast cancer (Liu et al., 2021). Although few similar strategies have been introduced to lung cancer, yet it has been reported that a stacked ensemble model has been established using cfDNA fragmentation features with superior sensitivity for detecting early-stage lung cancer with WGS data from cfDNA (Luo H et al., 2020).

Based on these successful attempts, we tried to explore the possibility of finding novel DNA methylation markers for LUAD from cfDNA methylome data. And our bisulfite PCR data from LUAD tissues and matched non-malignant tissues was highly consistent with the methylation feature of cfDNA, which further suggested the effectiveness of the strategy by combining the differential methylation profile between cfDNA from healthy people and LUAD patients to discover novel DNA methylation biomarkers. However, two of the seven CpGs, cg14780466, and cg14439622, were not validated by experiments because of technological constraints in PCR primers design. Our further study will continue to track the diagnostic and prognostic efficiencies of these seven CpGs associated with LUAD risk with high throughput technologies in a multicenter study for further validation. Nevertheless, the seven CpGs associated with LUAD risk are promising diagnostic and prognostic biomarkers for LUAD.

Additionally, the methylation states of DNA are universally altered in the early stage of various cancer types and such changes can be carried by cfDNA released from tumor cells (Feinberg, Ohlsson, and Henikoff, 2006). Hence, the detection of cfDNA methylation could be a promising approach for the early detection of various cancer types (Warton and Samimi, 2015). Similar strategies combined methylome analysis of cfDNA and tissues in cancer patients to identify less-invasive cancer-specific cfDNA markers have been reported in other cancer types, including breast cancer (Liu et al., 2021) and hepatocellular carcinoma (Li et al., 2021). It is reported that by utilizing specific analysis methods, ultrasensitive and robust cancer detection can be achieved by integrating DNA sequence and methylation information in plasma cfDNA from hepatocellular carcinoma (HCC) patients and healthy donors (Li et al., 2021). Not only cfDNA from plasma, but cfDNA from cerebrospinal fluid could be conducted to WGBS for discovering DNA methylation markers for medulloblastoma (Li et al., 2022). Together, these studies confirmed the feasibility of discovering DNA methylation markers by the joint analysis of cfDNA from plasma and genomic DNA from tissue for various cancer types, not only for LUAD.

In conclusion, the methylation levels of the 7-CpGs panel are promising biomarkers for diagnosing and predicting the OS of LUAD. And one of the seven CpGs, cg02261780, and its representing gene (GNA11) could not only be potential diagnostic and prognostic biomarkers, but also the potential therapeutic target of LUAD.

## Data Availability

The datasets presented in this study can be found in online repositories. The names of the repository/repositories and accession number(s) can be found in the article/Supplementary Material.

## Supplementary material

SUPPLEMENTARY FIGURE S1 GNA11 aberration analysis in LUAD. The data were downloaded from TCGA.

SUPPLEMENTARY FIGURE S2 Association between GNA11 expression and tumor stage. The data were downloaded from TCGA.

SUPPLEMENTARY FIGURE S3 Correlation analysis between gene expression and methylation of five CpGs (cg14780466, cg09595050, cg20193802, cg14439622, and cg05726109). The analyses were conducted with SMART across all transcriptome.

SUPPLEMENTARY TABLE S1 The original information of RRBS data from CFEA (23 lung cancers and 41 healthy donors) in the discovery cohort.

SUPPLEMENTARY TABLE S2 Survival analysis for each CpGs from Training cohort of TCGA.

SUPPLEMENTARY TABLE S3 DMC analysis in cell-free blood samples. DMC analyzed with cfDNA methylomes of lung cancers (n = 23) and healthy donors (n = 41) downloaded from CFEA detected by RRBS in the discovery cohort.

SUPPLEMENTARY TABLE S4 DMR analysis in cell-free blood samples. DMR analyzed with cfDNA methylomes of lung cancers (n = 23) and healthy donors (n = 41) downloaded from CFEA detected by RRBS in the discovery cohort.

SUPPLEMENTARY TABLE S5 The original information of RRBS data from CFEA (6 lung cancers and 33 healthy donors) in the validation cohort.

SUPPLEMENTARY TABLE S6 AUC analysis of the 7-CpGs methylation panel among different cancer types.
