# Supplementary figures and images for: Integrative analysis of DNA methylomes reveals novel cell-free biomarkers in lung adenocarcinoma
Source: Front Genet. 2023 Jun 16;14:1175784. doi: 10.3389/fgene.2023.1175784 (PMC10311559; doi:10.3389/fgene.2023.1175784)

# Expression of GNA11 in LUAD based on individual cancer stages

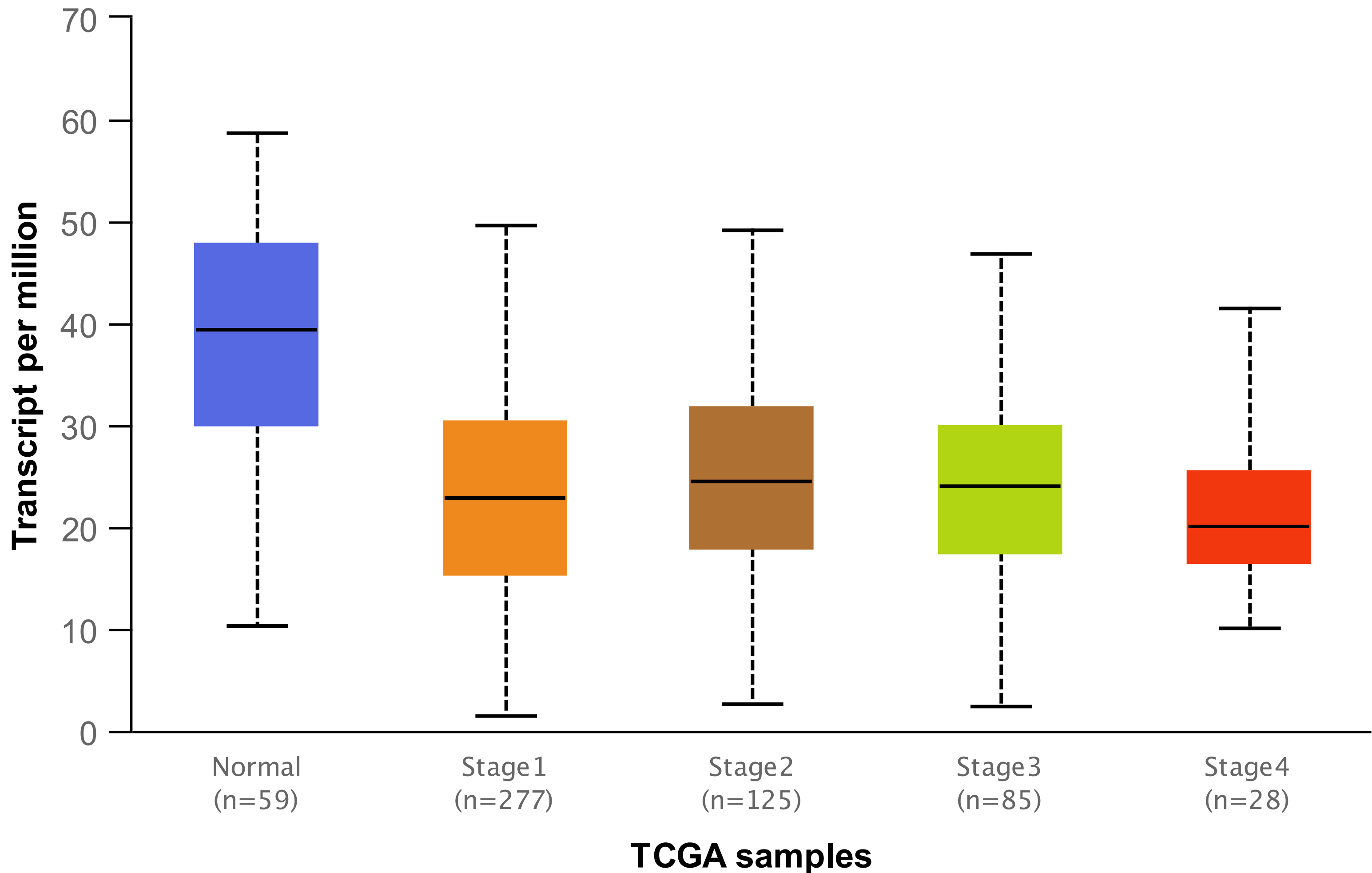

Supplement: Supplementary file 1 [file DataSheet2.PDF]

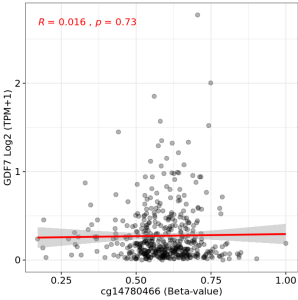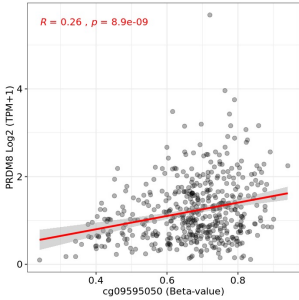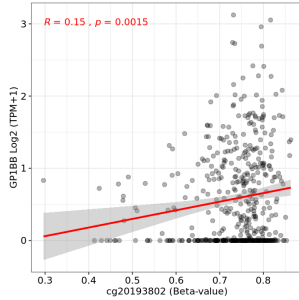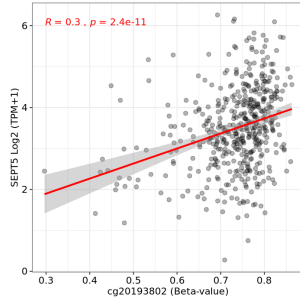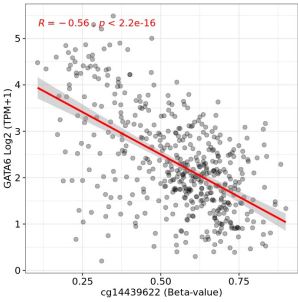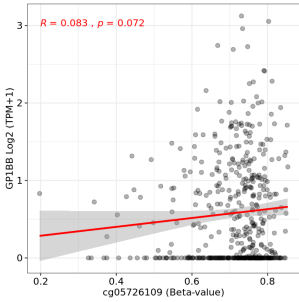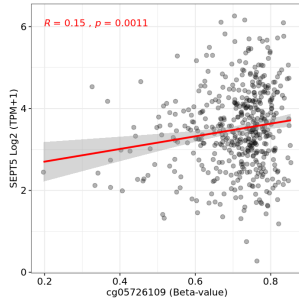

Supplement: Supplementary file 5 [file DataSheet3.PDF]

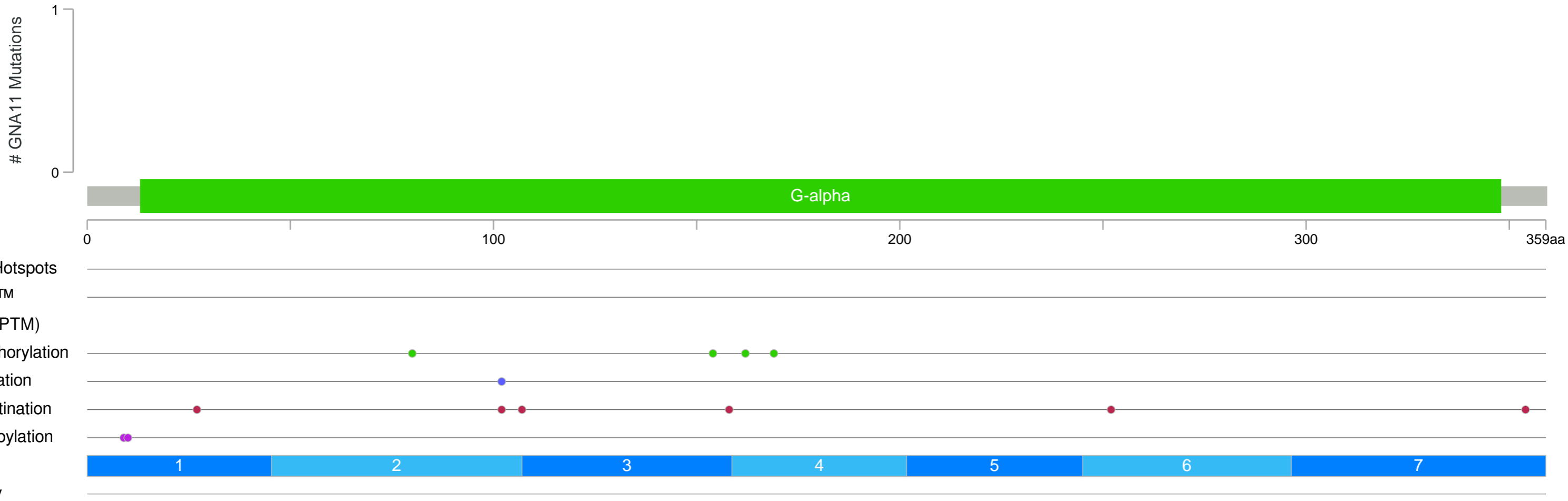

Supplement: Supplementary file 8 [file DataSheet1.PDF]
