# Supplementary material for: Integrative analysis of DNA methylomes reveals novel cell-free biomarkers in lung adenocarcinoma
Source: Front Genet. 2023 Jun 16;14:1175784. doi: 10.3389/fgene.2023.1175784 (PMC10311559; doi:10.3389/fgene.2023.1175784)
Supplement: Supplementary file 7 [file Table2.DOCX]

**Supplementary Table 2. Survival analysis for each CpGs in TCGA.**

| **Probe** | **Hazards. Ratio** | **CI. Lower** | **CI. Upper** | **p. value** | **Z** |
| --- | --- | --- | --- | --- | --- |
| **cg14780466** | **1.7762** | **0.3440** | **9.1713** | **0.4928** | **0.6859** |
| **cg02261780** | **7.0047** | **1.1998** | **40.8963** | **0.0306** | **2.1623** |
| **cg09595050** | **2.9596** | **0.7550** | **11.6015** | **0.1195** | **1.5568** |
| **cg20193802** | **0.6121** | **0.0913** | **4.1047** | **0.6132** | **-0.5056** |
| **cg14439622** | **3.7859** | **1.3233** | **10.8315** | **0.0131** | **2.4822** |
| **cg05726109** | **0.6760** | **0.1393** | **3.2818** | **0.6272** | **-0.4857** |
